# Capturing Unanticipated Drug Toxicities Using an Ensemble Machine Learning Approach

**DOI:** 10.21203/rs.3.rs-6999821/v1

**Published:** 2025-07-10

**Authors:** Nicole Zatorski, Avner Schlessinger

**Affiliations:** Duke University Hospital; Icahn School of Medicine at Mount Sinai

## Abstract

Despite rigorous safety evaluations during development, numerous drugs have been withdrawn from the market due to serious toxicities. Here we investigate the features found in drugs with these unanticipated toxicities and apply a machine learning approach to predict if a drug is likely to be withdrawn due to intolerable side effects without the need for human trial data. Our best preforming classifier was an ensemble predictor trained on protein targets, protein structure features, chemical fingerprints, and chemical features that achieved 92% accuracy and 0.845 Matthews Correlation Coefficient with 10-fold holdout test set cross validation. Analysis of features predictive of unanticipated toxicity revealed both known factors such as inhibition of cytochrome P450 as well as yet uninvestigated factors including the inhibition of bile salt export pumps. This predictor and subsequent feature analysis pave the way for the larger role of computational methods in screening potential candidates during drug development.

## INTRODUCTION

Unanticipated toxicities can emerge when drugs are incorporated into standard of care so numerous regulatory bodies monitor the safety of drugs for side effects after the end of their official clinical testing phase^1–4^. If unexpected side effects are observed, these agencies, such as the United States Federal Drug Administration (FDA) or the European Medicines Agency (EMA), can withdraw the drug from the market^1–4^. In addition to the impact unexpected toxicities have on patients, the decision to discontinue a drug further creates economic burden that prevents recoupment of research and development investment that increase costs of healthcare for patients^5^.

Due to the difficulties resulting from unexpected toxicities and subsequent drug withdrawals, many approaches have been used in an effort to predict toxicity earlier in the development pipeline^6^. Currently, the industry standard relies on laboratory and animal testing followed by Phase 1 trials in healthy volunteers to assess toxic side effects^7^. Traditional computation toxicity prediction approaches have centered around the use of drug Quantitative Structure-Activity Relationships (QSAR)^8^, which typically involve statistical or machine learning models that characterize the relationship between chemical properties of a drug and a phenotypes associated with toxicity. These features are used, for example, to predict a drug’s likelihood of inhibiting a particular protein, such as the hERG channel in the heart, which can precipitate arrhythmias when modulated by a drug^9^. While this system is fairly robust, it fails to capture the long-term use toxicities and very rare side effects uncovered in post marketing surveillance.

In addition, emerging big data analysis and machine learning techniques can be applied preclinically, during the drug development process, to provide a supplemental approach to toxicity prediction and eliminate potentially toxic drug candidates^10^. For example, deep neural network models applied to whole-genome DNA microarray data from the Open Toxicogenomics Project-Genomics Assisted Toxicity Evaluation System and DrugMatrix have predicted three liver toxicity endpoints with a range of 56–89% accuracy^11^. Cardio ToxCSM, is a webserver that predicts six cardiac toxicity outcomes from chemical drug structure representations and molecular descriptors achieving areas under the receiver operating characteristic curve (AUROC) ranging from 0.63 to 0.92^12^. ProTox-II provides predictions for 33 toxicity endpoints based on drug chemical data and 15 assayed targets^13^. These existing predictors, which are not yet used in established drug development pipelines, focus on detecting organ system or pathway toxicities rather than labeling drugs based on their likelihood of resulting in any side effects that necessitate withdrawal.

Structural features of protein drug targets have great potential for bolstering toxicity predictive models. Because these features describe the underlying structures of proteins, they are more readily able to capture similarities in different proteins^14^. These structural similarities, due to the interconnectedness of structure and function, therefore allow structural features to capture functional similarities and therefore similarities in drug effects. Structural features have been used, along with machine learning approaches, to predict protein-protein interactions^15^ and to construct genetic disease protein networks^16^. Protein structural features also capture patterns in normal human tissues^17^, cancer^14,18^, and drug perturbations^14,17^ from gene expression and proteomics data. These features therefore, combined with current machine learning techniques, hold great potential for revealing unanticipated drug toxicities.

Here we leverage data from 948 protein targets and their structural features with drug structural information to predict drug toxicity as defined by the compound’s withdrawal status. Drug withdrawal based on the recommendation of global drug safety regulatory agencies is a metric for drug toxicity that captures both extremely rare and severe drug side effects that have not been anticipated by traditional screening approaches. We begin with an examination of the different characteristics between withdrawn drugs and those still deemed safe to use. Indeed, through rigorous feature analysis, we identify drug and drug target features correlated with withdrawal drug status which can be used to inform future drug development. We then develop a predictor, named Withdrawal Anticipation and Toxicity Characterization of Hits (WATCH), to determine the likelihood of a drug having unanticipated toxicity leading to regulatory withdrawal. Furthermore, we discuss how WATCH can be applied as a screening tool to anticipate toxicities that may emerge in drugs under investigation by applying the predictor to drugs in clinical trials.

## RESULTS

### Drug withdrawals categorized by organ system

**Figure 1:**
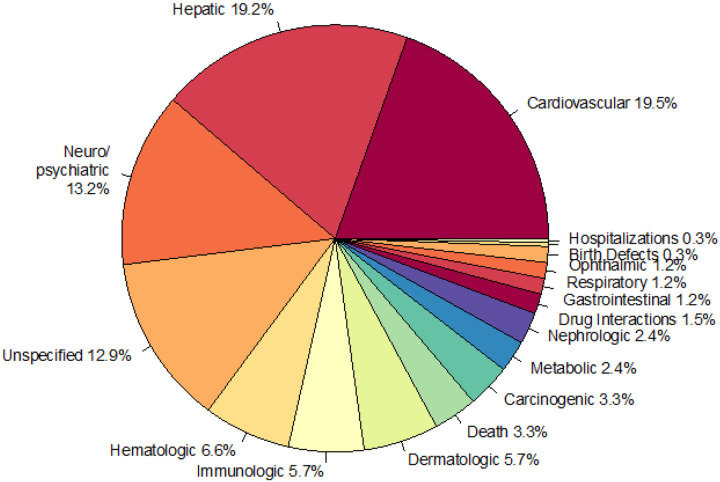
Causes of drug withdrawals listed by organ system of impact. Three broad toxicity types, including cardiovascular, hepatic, and neuropsychiatric comprise a majority of reasons for drug withdrawals. Data was compiled from the ChEMBL database (Gaulton, et al., 2017). A single drug can have more than one potential cause for withdrawal.

The 120 drugs, found in the ChEMBL database^19^, that have been withdrawn by regulatory agencies worldwide have targeted a variety of organ systems (Supplementary Fig. 1). Interestingly, side effects affecting three organ systems make up a majority of reasons for drug withdrawal from the market. Cardiovascular (19.5%), hepatic (19.2%), and neuropsychiatric toxicity (13.2%) are the three largest contributors to drug withdrawal (Fig. 1). Cardiovascular reasons for withdrawal are largely due to arrhythmias and conditions that predispose to arrhythmias: arrhythmias (12%), QT prolongation (6%), ventricular tachycardia (6%), torsade de pointes (6%), ventricular arrhythmias (5%), and repolarization abnormalities (3%) (Supplementary Fig. 2A). This is supported by the focus of early cardiotoxicity screens, which primarily investigate compound interactions with arrhythmia inducing channels such as hERG^9^. Besides general hepatotoxicity, liver failure is the second largest listed reason for hepatotoxicity (8%) (Supplementary Fig. 2B). In contrast to hepatotoxicity and cardiotoxicity, neuropsychiatric toxicity has more variety of side effects that range from abuse (21%) to central nervous system stimulation (2%) (Supplementary Fig. 2C). This reflects the wide range of observed neuropsychiatric toxicities and the difficulty in the development of *in vivo* screening tests which can be applied prior to lengthy animal studies^20^. Neuropsychiatric drugs are well known for their polypharmacology, where individual drugs bind many proteins, which contributes to increased frequency and severity of toxicity^21^.

### Comparison between withdrawn and not withdrawn drugs by indication

**Figure 2:**
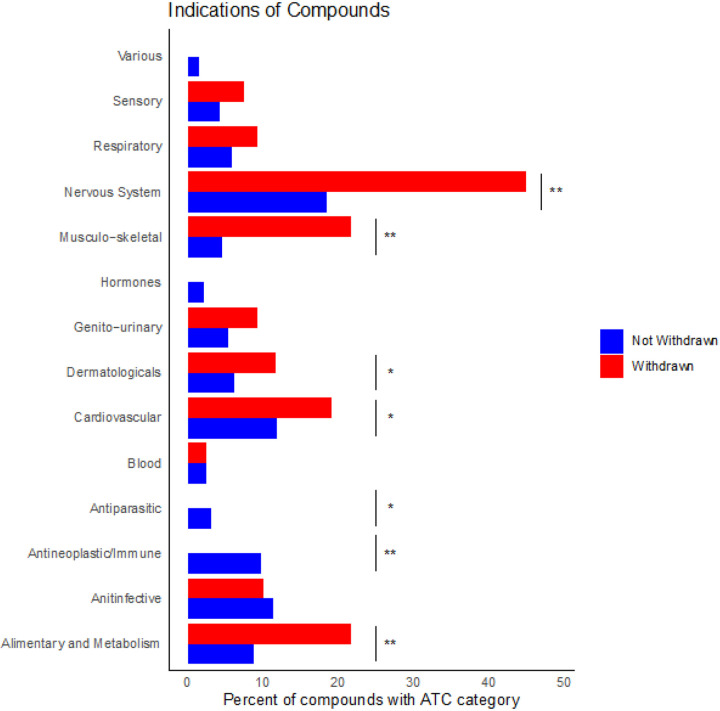
Indication of withdrawn drugs compared to indications of approved drugs. Indication are defined based on the first letter (Level 1) of the ATC code. Percent of compounds with that ATC first letter designation compared to the total number of compounds in that category is shown on the horizontal axis. Pairwise comparisons between frequencies of ATC indications performed with a fisher exact test show the following indications have differences between withdrawn and not withdrawn drugs: anti-infective, antineoplastic/immune, antiparasitic, hormones, musculo-skeletal and nervous system. A p-value lower than 0.05 is indicated by a *, and a p-value lower than the Bonferroni corrected significance level is indicated by a **.

A fisher exact test analysis of drug Anatomical Therapeutic Chemical (ATC) codes^22^, a hierarchical classification system of medicines maintained by the World Health Organization (WHO), reveals differences in target organ system and disease indication between drugs that are in use and those that have been withdrawn (Fig. 2). Withdrawn drugs have different frequencies of therapeutic indications from drugs that are not withdrawn for various treatment types. For example, neither the antineoplastic nor antiparasitic categories have any withdrawn drugs. Antineoplastics account for 10% of not withdrawn drugs (p = 1.66e-5) and antiparasitic drugs comprise 3% of not withdrawn drugs (p = 0.0448) (Fig. 2). For other ATC indications, more drugs have been withdrawn than not withdrawn. Drugs that are indicated for diseases of the alimentary and metabolic systems (22% of withdrawn and 9% of not withdrawn p = 3.63e-5), cardiovascular system (19% of withdrawn and 12% of not withdrawn p = 0.0294), dermatologic system (12% of withdrawn and 6% of not withdrawn p = 0.0331), musculo-skeletal system (22% of withdrawn and 5% of not withdrawn p = 4.40e-10), and nervous system (45% of withdrawn and 18% of not withdrawn p = 1.90e-10) have a higher frequency of withdrawals. A sub analysis of drugs actively in clinical trials reveals that the primary indications of focus are: alimentary and metabolism (16%), antineoplastic and immune (13%), cardiovascular (12%), and nervous system (13%).

### Features of withdrawn drugs and their protein targets

**Figure 3:**
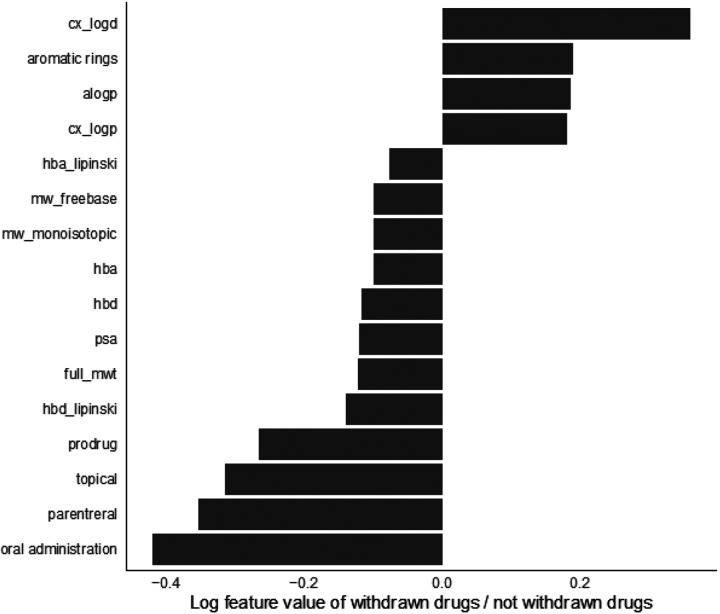
Drug chemical features of withdrawn drugs compared to not withdrawn drugs. The average values of continuous variables and the frequency counts normalized by the total number of drugs in each category for categorical variables were calculated for withdrawn and not withdrawn drugs. For each statistically significantly different chemical feature variable, the log base 2 value of the ratio of the withdrawn drug divided by the not withdrawn drug was plotted. Features with positive values are larger in withdrawn drugs than not withdrawn drugs. All features shown have a p-value smaller than 0.05. Feature names correspond to those found in ChEMBL chembl_webresource_client. Features that are over represented in withdrawn drugs include lipophilic characteristics (cx_logd, alogp and cx_logp, which correspond to logD and logP respectively) as well as number of aromatic rings. Features that are under represented in withdrawn drugs include the number of atoms participating in hydrogen bonds (hydrogen bond acceptors and donors- hba, hbd, hba_lipinski, hbd_lipinski), the molecular weight (mw_freebase, mw_monoisotopic, full_mwt), polar surface area (psa), status as a prodrug, and routes of administration (topical, parenteral, and oral administration).

To determine the characteristics that mark a drug removed from use due to toxicity, we investigated drug structural and pharmacological features through recursive feature elimination with cross validation (RFECV)^23^ using a random forest model, variance threshold, and generic univariate selection (Methods). Feature selection of drug chemical features demonstrates that route of administration, formulation as a prodrug, acidity (as measured by apKa), quantitative estimate of drug-likeness^24^, drug identity as a base, number of hydrogen bond acceptors (hba), molecular weight (MW), polar surface area (tPSA), lipophilicity (i.e. logP, logD), and frequency of heavy atoms are all highly distinguishing features between withdrawn and not withdrawn drugs (Fig. 3, Supplementary Table 1). Consistent with these results, underrepresented features in withdrawn drugs include molecular weight, frequency of heavy atoms, polar surface area, and quantitative estimate of drug-likeness (Fig. 3).

We next applied the feature selection approach to the protein targets, including the protein name, as well as protein structural and functional features, such as secondary structure elements, disordered regions, and coiled-coils. These proteins include intended and unintended targets of the drug. The most informative drug targets include proteins involved in drug absorption, disposition, metabolism, and excretion (ADME) that are screened in drug development as part of drug toxicity target panels, such as the metabolic enzyme Cytochrome P450 (3A4, 2C19, 2J2)^25^; and the membrane transporters^26,27^ the ATP-binding cassette sub-family C (members 2–4), the bile salt export pump (ABCB11), SLC family 22 member 1 (SLC22A1, OCT1), multidrug and toxin extrusion protein 1 (SLC47A1, MATE1), and the ATP-dependent translocase ABCB1 (Table 1, Supplementary Table 3). The feature selection paradigm also uncovers proteins not regularly screened by for activity during drug development, such as the sodium dependent dopamine transporter DAT (SLC6A3). Feature analysis of the structural characteristics of the proteins inhibited by drugs indicate an importance in composition of helical regions, turn regions, and number of amino acid contacts (Supplementary Table 3).

**Table:** 

Protein Target^A^	Gene Name^B^	Protein Target Name^C^	Biological Function^D^	Drugs with lowest IC_50_ standard values^E^
O15438	ABCC3	ATP-binding cassette sub-family C member 3	transports drugs across cell membranes	Cyclosporin A, amg-009, olmesartan medoxomil
O95342	ABCB11	ATP-binding cassette sub-family B member 11	transports bile salts across hepatocyte membrane	Alisporivir, BDBM172715, Pioglitazone
O15439	ABCC4	Multidrug resistance-associated protein 4	transports compounds out of cells	Estradiol 3,17-disulfate, Tranilast, Sulfasalazine
P08684	CYP3A4	Cytochrome P450 3A4	mediates metabolism of steroid hormones	BDBM144637, BDBM50448963, BDBM144636
Q92887	ABCC2	ATP-binding cassette sub-family C member 2	transports drugs across cell membranes	Leukotrien C4, Bilirubin monoglucuronide, Bilirubin bisglucuronide
O15245	SLC22A1	Solute carrier family 22 member 1	transports organic cations	chlorhexidine, SKF550, Decynium-22
Q96FL8	SLC47A1	Multidrug and toxin extrusion protein 1	transports cationic compounds	ondansetron, imatinib, ritonavir
P33261	CYP2C19	Cytochrome P450 2C19	mediates metabolism of fatty acids	Sulconazole, loratadine, Miconazole
Q01959	SLC6A3	Sodium-dependent dopamine transporter	mediates dopamine transport	hydroperoxycadiforin, Rhenium complex, BAY-390
P51589	CYP2J2	Cytochrome P450 2J2	mediates metabolism of fatty acids	Chlorzoxazone, Danazol, BDBM235143

### Prediction of drug withdrawal status

**Figure 4:**
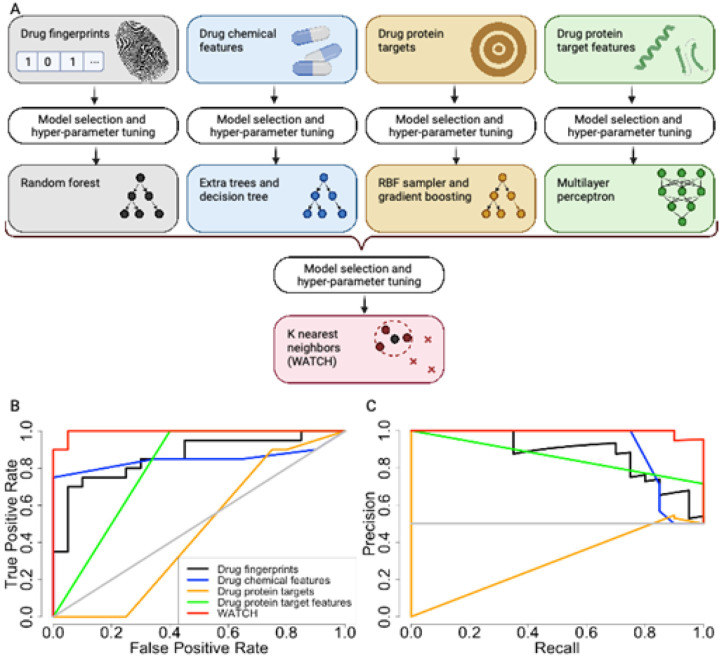
Model predicting drug withdrawal performance on representative test set. Top-performing classifiers are shown for each data set, and are as follows: multilayer perceptron for drug target features, RBF sampler for drug targets, extra trees classifier stacked with a decision tree for drug chemical features, random forest for drug fingerprints, and K-nearest neighbor for the ensemble. A. Model architecture for the withdrawn drug predictor. Each data set was divided into training, test, and validation sets. The training set was used to select and tune the hyperparameters for separate models. The test set was used to evaluate model performance. Predictions from these models were combined to form a new data set which served as the training data for the meta predictor. The ensemble predictor was trained on the pooled predictions from the individual predictors using the original training set and the test set combined. The model was evaluated using validation set. B. Receiver Operating Characteristic (ROC) curve showing predictive model true positive rate compared to false positive rate for each of the models based on different input data as well as the overall ensemble predictor. C. Precision-recall curves for each of the models based on different input data as well as the overall ensemble predictor. Using the two different evaluation measures, the ensemble-based predictor preformed the best of all models.

We hypothesized that these differences between withdrawn and not withdrawn drugs can be exploited to predict if a drug is likely to be withdrawn by regulatory agencies due to unanticipated toxicity using a machine learning. We developed various classifiers trained on protein targets, protein target structural features, as well as molecular fingerprints of the drug, and other structural features of drugs. Models were selected and optimized for each dataset separately. Top models were determined by maximizing the sum of model accuracy, F1, Precision, Recall, and MCC. The various datasets, which each contain different types of features, individually display a wide range of performances (Fig. 4BC). Of the individual predictors, the classifier trained on inhibited protein features, a multilayer perceptron, performed the best with an accuracy of 84.3%, F1 of 0.867, and an MCC of 0.725 (Table 2). The classifier trained on drug features, a stacked extra trees and decision tree, performed with an accuracy of 77.0% (Table 2). The classifier trained on molecular fingerprints, classified with random forest, and inhibited proteins, classified with an RBF sampler and gradient boosting, performed with accuracies of 68.3% and 62.8% respectively (Table 2).

The predictions from the individually trained classifiers were combined using an ensemble predictor, the Withdrawal Anticipation and Toxicity Characterization of Hits (WATCH) (Fig. 4A). To minimize overfitting, holdout data was used in the training of WATCH (Methods). Application of this ensemble learning approach improved performance of drug prediction of withdrawal status compared to individual models trained on chemical fingerprints, chemical features, inhibited proteins, and features of inhibited proteins respectively (Fig. 4BC).

Overall accuracy of this K nearest neighbor classifier on the balanced test data set was 92.0%, F1 was 0.919, and MCC was 0.845 (Table 2). For comparison, averaging the individual model predictions achieved accuracy of 85.5% and a MCC of 0.720 (Table 2). Therefore, WATCH demonstrated improved performance compared to the best individual classifier and the simple averaging of all the predicted probabilities from each individual model. Thus, using a combination of feature types derived from drug chemical structure as well as protein targets, WATCH was best able to predict withdrawal status of a compound of all tested models.

**Table:** 

Data	Model	Accuracy	F1	Precision	Recall	MCC
Ensemble of all data sets	K nearest neighbors	0.920	0.919	0.929	0.915	0.845
Averaged prediction of all models on each data set	Average	0.855	0.859	0.839	0.890	0.720
Features of protein targets	Multilayer perceptron	0.843	0.867	0.769	1.000	0.725
Protein targets	RBF sampler and gradient boosting	0.628	0.704	0.589	0.880	0.294
Molecular fingerprints	Random forest	0.683	0.690	0.680	0.710	0.370
Drug features	Stacked extra trees and decision tree	0.770	0.766	0.850	0.750	0.581

### Drug withdrawal status predictor applied to drugs in clinical trials

**Figure 5:**
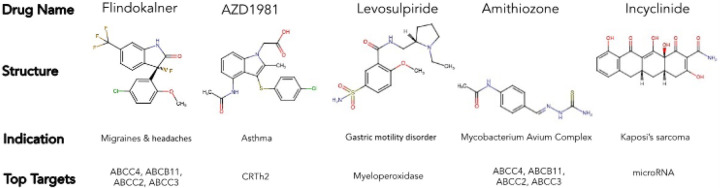
Prediction of withdrawal of drugs in clinical trials. The predictor was trained on 10 different balanced random subsets of existing withdrawn and not withdrawn drug data. These independently trained models were applied to new drugs currently in clinical trials to assess future withdrawal status of these candidate compounds. This figure shows structures of compounds that are currently in clinical trials and are predicted to be withdrawn by the predictor in 10 out of 10 prediction iterations. These include three compounds with serious known toxicities and toxic targets.

Once WATCH was trained and validated on drug data collected it was tasked with predicting the likelihood that drugs in investigative trials at that time which would ultimately be withdrawn by regulatory agencies due to toxicity. Certain drugs in phase 1–3 clinical trials^19^ were predicted to belong to the withdrawn drug class (Supplementary Table 4). Following ten iterations of prediction, a majority of compounds were not predicted to be withdrawn (4 withdrawal predictions or less) (Supplementary Table. 4). Just over 25% of drugs were labeled as likely to be withdrawn in 50% of iterations. There were 5 drugs however that were predicted to be withdrawn in each of the ten iterations: Flindokalner, Levosulpiride, Amithiozone, AZD1981, and Incyclinide (Fig. 5).

## DISCUSSION

Drug withdrawal from the market is disruptive to patient care and drug development. Regulatory agencies decide to withdraw a drug after serious and unexpected toxicities in patients are observed. These rare or late manifesting toxicities have not been detected during the normal rigorous drug design process. Here we characterize withdrawn drugs using various features and apply this knowledge in combination with an ensemble predictor to anticipate drug withdrawal status earlier in drug development.

Our study provides an overview for landscape of withdrawn drugs. Side-effects associated with withdrawn drugs (derived from ChEMBL) primarily affect three organ systems including the liver, the cardiovascular system, and the neuropsychiatric system (Fig. 1). This is consistent with multiple studies investigating drug toxicity^28,29^. Moreover, comprehensive analysis of ATC codes (Fig. 2), enabled comparisons of trends in withdrawn and non-withdrawn drugs. In the case of antineoplastic drugs, underlying disease severity and resistance development may influence withdrawal status. For example, although vincristine is associated with serious neuropathy, it is still an important part of the regime used to treat childhood cancers such as acute lymphoblastic leukemia, Hodgkin lymphoma, rhabdomyosarcoma, nephroblastoma, medulloblastoma, and low-grade glioma^30^. Similarly, the potential for multidrug resistance increases the need for numerous approved antineoplastic therapies in order to allow for multidrug therapies^31^. Antiparasitic drugs also have no withdrawn treatments. However, in contrast to antineoplastic drugs, the shorter treatment course of certain antiparasitic drugs-less than 90 days^32^- is hypothesized to play a role in this trend. The high number of withdrawn drugs in other categories could potentially result from the number of other existing treatments for diseases in these categories combined with a different assessment of a toxicity to benefit ratio^33^. Taken together, this analysis suggests the possible underlying influence of treatment course and disease severity on withdrawal status, as well as demonstrates current priorities in research and development strategies.

To gain more insight into what makes a drug toxic, we characterized drugs based on their chemical and pharmacological features. Feature selection using a combination of three methods distinguished important characteristics of globally withdrawn drugs that can be employed in future drug development. Interestingly, the top four chemical features overrepresented in withdrawn drugs as compared to drugs still in use are related to drug lipophilicity. In particular, logP and logD could be informative because they capture drug distribution throughout the body, with higher concentrations of drug in more locations increasing the potential for binding off-target increasing side effects. Consistent with these results, underrepresented features in withdrawn drugs include molecular weight, frequency of heavy atoms, polar surface area, and quantitative estimate of drug-likeness, which also affect the drug’s ability to cross lipid membrane bilayers, translating to drug oral bioavailability^24,34^. Route of administration, another set of features extracted by this analysis, is vital for the understanding of the speed and level of systemic reach of a compound upon use. Indeed, chemical features highly correlated with drug withdrawal including route of administration and pKa reflect known pharmacological ADME design trends (Fig. 3). These features can be used in the chemical design process and provide additional confidence in our approach as they overlap with existing drug chemical structure rules^24,34^. In the future, drugs with properties that have values in the withdrawn range may become candidate for more thorough screening or may be altered chemically during early stages of development.

Similarly, our feature selection approach highlighted the importance of certain drug proteins targets, some of which have known and experimentally validated toxicity. Cytochrome P450 (3A4, 2C19, 2J2), one of the most informative proteins corelated with toxicity according to our feature selection paradigm, is well established as a toxic drug target due to its ability to modulate the metabolism of other drugs^25^, involvement in drug-drug interactions^35^ and pharmacogenetics^36^. Certain membrane transporters, which often have similar pharmacological role to that of metabolic enzymes in controlling drug ADME^26^, are also implicated in toxicity (Supplementary Table 2). For example, drug modulation of the ATP-binding cassette sub-family results in cardiotoxicity^37^. The bile salt export pump (ABCB11) has known association with hepatotoxicity^38^. Interestingly, the sodium dependent dopamine transporter DAT (SLC6A3), which is targeted by many CNS drugs such as SSRIs and SNRIs, was also correlated with toxicity in our study. This transporter is not known to be related to pharmacokinetics, and thus, it is not regularly screened by for activity during drug development like the drug ADME transporters above. Features of these proteins such as composition of helical regions and number of amino acid contacts also play a large role in the pattern of drug toxicity further explored by our predictive machine learning model. Interestingly, these features are commonly observed in membrane proteins, such as the transporters discussed^39^. Our work therefore suggests additional proteins and structural features of proteins which would be beneficial to include in drug toxicity screening panels.

To utilize our new insights on drug toxicity, we developed a screening tool for flagging potentially toxic drugs. We built an ensemble predictor (Fig. 4A), Withdrawal Anticipation and Toxicity Characterization of Hits (WATCH), which applies classifiers to various chemical and protein features to label a drug with its withdrawal potential. The 10-fold cross-validated ensemble predictor assessed on balanced, holdout test sets scored an accuracy of 92.0%, F1 of 0.919, and a MCC of 0.845 (Table 2).

We also analyzed the clinical trial drug landscape and applied the WATCH predictor to these compounds. The indications of clinical trial drugs reflect a slight predominance of new drugs targeting the alimentary and metabolism (16%) system. (Supplementary Fig. 3). Additional indications common in drugs currently in clinical trials include antineoplastic and immune (13%), cardiovascular (12%), and nervous system (13%) drugs. With the exception of antineoplastic drugs, which do not have as many drugs available as these other groups, these are all areas in which more drugs have been withdrawn than are currently available (Fig. 2). The preponderance of withdrawn drugs in these categories points to unmet needs that new drugs are being designed to fill. WATCH applied to drugs in clinical trials distinguished a number of drugs that are expected to be withdrawn from the market (Fig. 5). Interestingly, Flindokalner targets potassium ion channels^40^, which overlaps with the know toxic targets found through recursive feature elimination, and is indicated for migraines and post-traumatic headaches (NCT03887325). Levosulpiride is indicated for gastric motility disorders. In trials this drug has been observed to cause severe side effects such as acute dystonias and Parkinson like symptoms^41^. Amithiozone has been used as a treatment for tuberculosis, but was replaced with newer, less toxic antibiotics^42^. Currently, Amithiozone is being re-investigated as a therapy for Mycobacterium Avium Complex^43^. AZD1981 is a CRTh2 receptor antagonist indicated as an asthma therapy^44^. Incyclinide is an antineoplastic agent^45^. In summary, many of the drugs flagged by the withdrawn prediction model, have already begun to demonstrate serious side effects in clinical trials. This demonstrates the applicability of the predictor for future drug toxicity investigations. Furthermore, the characterization of withdrawn drugs and WATCH are meant to serve as tools for drug developers in the ever-evolving endeavor to predict the toxicity of candidate compounds.

## METHODS

### Drug ATC analysis

Using the withdrawal reasons and ATC datasets, we analyzed the main organ systems that drugs target. We manually grouped the non-standardized labels describing reasons for drug withdrawal (Fig. 1, Supplemental Fig. 2). In addition, to investigate the differences between organ systems of action in withdrawn and not withdrawn drugs, we used the anatomic therapeutic chemical (ATC) code^22^ designation, the first letter of which indicates anatomical main group^46^. Frequencies of withdrawn and not withdrawn drugs for each therapeutic indication category were compared using the scipy.stats.fisher_exact test 1.6.0 (Fig. 2). Drugs without an ATC code were omitted. The Bonferroni^47^ adjusted p value was 0.0036. It is possible for a drug to have more than one reason for withdrawal and organ system of indication.

### Feature selection

Characteristics that most distinguished between withdrawn and not withdrawn drugs were determined with three different feature selection methods on three sets of data features (drug molecular features, drug targets, and drug target features). Specifically, the methods we used were: (i) recursive feature elimination with cross validation (RFECV) in scikit learn 1.1.2. The cross validation splitting strategy (cv) equaled 5 folds, the steps value equaled 1, and the classifier was a random forest model; (ii) Scikit learn 1.1.2. variance threshold with a threshold of 0; and (iii) generic univariate selection for the top 25% of features with a chi squared score function was also applied. These three feature selection methods were applied to each dataset separately 10 times using a random balanced 80% of the data each time and random seeds 0–9. Scores were normalized to be out of 10, where the best features would score a 10, and combined for each feature selection model with equal weights.

For visualization of the drug chemical features, the average values of continuous variables and the frequency counts normalized by the total number of drugs in each category for categorical variables were calculated for withdrawn and not withdrawn drugs. For each statistically significantly different chemical feature variable, the log base 2 value of the ratio of the withdrawn drug divided by the not withdrawn drug was plotted (Fig. 3).

### Prediction of compound withdrawal status

Drug withdrawal status for existing drugs was predicted using a variety of machine learning approaches. First the fingerprint, drug chemical features, target, and target feature datasets were used independently to generate predictions. Each of the datasets were randomly split into balanced 80-10-10 percent segments. These represent the training, first test, and second test sets respectively. Balancing of the sets ensured an equal number of withdrawn and not withdrawn drugs in each training and testing tranche. Each feature was min-max normalized (Eq. 1) using the minimum and maximum values of the feature in the training set. The Tree-based Pipeline Optimization Tool (TPOT)^48^ 0.11.7 was used to tune hyperparameters for TPOT neural networks, scikit learn random forest, scikit learn multilayer perceptron, and xgboost extreme gradient boosting. The parameters for TPOT were: population size of 24, offspring size of 12, early stop of 12, scoring using accuracy, cv of 5, and generations of 5. The parameter search space for the models are as follows. Random forest: criterion was entropy or gini; max depth was none and 10 through 500; max features were auto, square root, log2 or none; min samples per leaf were 2 through 15, min sample split was 5 through 15; number of estimators were 150 through 500. Multilayer perceptron: activation was identity, loginst, tanh, or relu; hidden layer sizes were 25 through 400; solver was lbfgs, stochastic gradient descent, or adam. Extreme gradient boosting: max depth was none or 10 through 500; number of estimators was 150 through 500. Following hyperparameter tuning, the top performing model was selected for each data set.

Accuracy, area under the receiver operating characteristic curve (AUROC), F1, precision, recall, and Matthews Correlation Coefficient (MCC) were calculated with scikit learn on the first test set.


Equation 1 $$\text{normalization}=\frac{\text{value}-\text{min}(\text{feature}\;\text{values})}{\text{max}(\text{feature}\;\text{values})-\text{min}(\text{feature}\;\text{values})}$$

The output of the best overall performing model for each of the 4 datasets was used to generate the training and test sets for the meta classifier, Withdrawal Anticipation and Toxicity Characterization of Hits (WATCH). These were the models that maximized the sum of the accuracy, AUROC, F1, precision, recall, and MCC. The training set consisted of the training set for the previous model in addition to the test set for the first model. The test set was the second holdout test set from the initial data split. Hyperparameter tuning and model selection for the meta classifier proceeded as previously described. The best meta classifier model, WATCH, was a K nearest neighbor classifier with 57 neighbors, and distance weights. This was compared to a meta classifier model that averaged the predictions for the best models on the 4 datasets. Accuracy, area under the receiver operating characteristic curve (AUROC), F1, precision, and recall were calculated with scikit learn on the second, holdout test set.

The best performing classifier for the target structural feature dataset was a multilayer perceptron with identity activation, 150 hidden layers, and a stochastic gradient decent solver. The best performing classifier for the proteins was a RBF sampler with 0.05 gamma and a gradient boosting classifier with 0.1 learning rate, 9 max depth, 0.65 maximum features, 14 minimum samples per leaf, 15 minimum samples per split, 100 estimators, and 0.5 subsamples. The best performing classifier for the drug chemical features dataset was an extra trees classifier with bootstrap, gini criterion, 0.65 maximum features, 7 minimum samples per leaf, and 100 estimators stacked with a decision tree with gini criterion, maximum depth of 5, 18 minimum samples per leaf, and a minimum samples per split of 3. The best performing classifier for the drug fingerprint dataset was a random forest classifier with entropy criterion, 64 maximum depth, log2 maximum features, 2 minimum samples per leaf, 5 minimum samples per split, and 188 estimators. (Fig. 4BC)

### Prediction of toxicity for drugs under clinical investigation

We applied WATCH to existing drugs to drugs currently in clinical trials (1216 drugs). The classifier selected for WATCH was the K nearest neighbor meta classifier, which maximized the sum of the accuracy, AUROC, F1, precision, recall, and MCC. WATCH, run 10 times with random states 0–9, was used to classify all drugs found in ChEMBL currently in phase I-III clinical trials (downloaded using chembl-webresource-client 0.10.8 on January 16, 2023). The output for the 10 runs of WATCH for each drug was summed. The closer the resulting value a drug had to 10, the more likely it is to be withdrawn in the future.

## DATA AVAILABILITY

### Drug datasets

We constructed four drug feature datasets, including drug withdrawal status dataset, drug indication dataset, drug fingerprint dataset, and drug feature dataset. The withdrawal status dataset contains drug withdrawal status and reasons for drug withdrawal collected from ChEMBL^19^ using chembl-webresource-client 0.10.8. Of the 230 drugs listed as withdrawn, 120 had reasons for withdrawal found in the database. We manually curated these non-standardized reasons for withdrawals into logical uniform labels (for example “Hepatic toxicity” and “Hepatotoxicity” were combined under the label of Hepatotoxicity). We further grouped each toxicity by the organ system most effected (for example Liver for Hepatotoxicity).

The drug indication dataset contains the anatomic therapeutic chemical (ATC) codes, which classifies drugs by site of action and chemical characteristics^35^, for withdrawn (n = 120) and not withdrawn drugs (n = 1511) also determined using ChEMBL^19^.

The drug fingerprint dataset contains Morgan fingerprints^49^ with radius 2 and 512 bits as vectors generated with Rdkit 2022.09.1 for withdrawn and not withdrawn drugs.

The drug feature dataset contains chemical features of withdrawn and not withdrawn drugs found in ChEMBL^19^. The molecular features included in this dataset are: chirality, partition coefficient (alogp (calculated), cx_logp (generated with ACDlabs v12.01)), number of aromatic rings, full molecular weight (including salts), freebase weight (weight of parent compound), and monoisotopic weight (weight of the monoisotopic parent compound), route of administration (oral, parentreral, or topical), status as a prodrug, distribution coefficient (cx_logd (generated using ACDlabs v12.01), the most acidic acid dissociation constant (cx_most_apka), most basic acid dissociation constant (cx_most_bpka), number of hydrogen bond acceptors (hba, hba_lipinski), number of hydrogen bond donners (hbd, hbd_lipinski), number of heavy atoms, identity as an acid, identity as a base, identity as a neutral molecule, identity as a zwitterion, drug likeness (num_lipinski_ro5_violations (number of rule of 5 violations using Lipinski hydrogen bond acceptor and donor definitions), num_ro5_violations (number of rule of 5 violations using hydrogen bond acceptor and donor definitions), qed_weighted (quantitative estimate of drug likeness^24^)), polar surface area (psa), rule of three (ro3_pass^50^), and number of rotatable bonds (rtb).

### Drug target datasets

We also constructed two drug target feature datasets, including IC_50_ values dataset, and drug target features. The drug target dataset contains IC_50_ values for each human protein drug target found in ChEMBL^19^.

The drug target features were derived from the amino acid sequence and structural models of the proteins in the drug target dataset (Supplementary Table 3). The method for feature generation has been previously described^14^. In brief, structural features, such as protein secondary structure or intrinsically disordered regions, describe characteristics of protein regions. These structural features are determined from either protein sequence, using the methods IUPRED2a^51^, ANCHOR^52^, TMHMM 2.0^53^, PredictProtein^54^, SCOPe^55^; or from three-dimensional model predicted with AlphaFold2^56^, using the methods Dictionary of Protein Secondary Structure (DSSP)^57^, biopython’s pdb parser^58^, Aggrescan3d^59^. Cutoffs for classifying region identity have been determined in previous work^14^. Features for each protein were normalized by the length of protein and then weighted according to Eq. 2. Where F is the normalization value of the protein feature, f is the unnormalized value of the protein feature, sIC_50_ is the set of IC_50_ values for the protein tested with all drugs, and pIC_50_ is the IC_50_ of the protein being normalized.


Equation 2 $$F=f\frac{\text{max}(\text{sI}C_{50})-\text{pI}C_{50}+1}{\text{max}(\text{sI}C_{50})}$$

## Supplementary Files

This is a list of supplementary files associated with this preprint. Click to download.

- SupportingInformationFinal.docx

## Data Availability

Data and code can be found in the GitHub repository: https://github.com/schlessinger-lab/withDRAWN.

## CODE AVAILABILITY

A total of 1,296 compounds (204 withdrawn and 1,092 not withdrawn) were found having fingerprints, targets, target features, and drug molecular features. A full list of drugs and their features can be found in the project GitHub repository: https://github.com/schlessinger-lab/withDRAWN.
